# Improving the Psychosocial Work Environment to Prevent Sickness Absence and Turnover in Nurses: A Systematic Review

**DOI:** 10.1155/jonm/7860752

**Published:** 2025-10-22

**Authors:** Laurent Corthésy-Blondin, Alessia Negrini, Samantha Vila Masse, Christine Genest

**Affiliations:** ^1^Institut de Recherche Robert-Sauvé en Santé et en Sécurité du Travail, 505 Boul. De Maisonneuve Ouest, Montréal, Québec H3A 3C2, Canada; ^2^Faculty of Nursing, Université de Montréal, 2375, Chemin de la Côte-Ste-Catherine, Montréal, Québec H3T 1A8, Canada; ^3^Centre de Recherche de l'Institut Universitaire de Santé Mentale de Montréal, 7401 Rue Hochelaga, Montréal, Québec H1N 3M5, Canada

## Abstract

In nurses, the high rates of sickness absence (SA) and turnover generate staggering costs. Their prevalence could be reduced by acting on the work-related psychosocial factors (WRPFs) that can impact the mental health of nurses and lead to their absences and departures. However, there is a lack of consensus regarding the effectiveness of interventions that target WRPFs from a SA and turnover prevention perspective. This systematic review aims to identify interventions that target WRPFs to reduce SA and turnover in nurses, describe the methods used to evaluate their effectiveness, and report on their effectiveness at improving SA, turnover rates, and turnover intention. A systematic search was conducted using eight online databases and search engines (i.e., CINAHL, Embase, Catalogue ISST, Google Scholar, OSH Update, PsycINFO, PubMed, and Social SciSearch). Empirical studies that focus on an intervention that targets at least one WRPF and aims at reducing SA, turnover rates, or turnover intention among nurses were included. The methodological quality of each study was assessed using the Medical Education Research Study Quality Instrument. Fourteen articles focusing on 13 interventions met the inclusion criteria. The interventions targeted individuals (*n* = 4), groups (*n* = 2), leaders (*n* = 2), and organizations (*n* = 5). The interventions aimed mainly at reducing workplace bullying and lateral violence and improving leadership. The research designs varied greatly across studies, and the results regarding the effects of the interventions on SA and turnover behaviors and intention were inconsistent. Innovative interventions targeting WRPFs need to be developed and implemented, and sophisticated methods to evaluate their effectiveness at reducing SA and turnover behaviors and intention should be employed.

## 1. Introduction

Sickness absence (SA) and turnover are salient issues in the healthcare sector [1, 2]. SA refers to an involuntary and unplanned absence from work due to a health problem experienced by the worker, for at least one day within a defined period [3, 4]. Turnover refers to being voluntarily transferred (e.g., to another position or department) while remaining in the same organization (internal turnover) or voluntarily leaving one's organization (external turnover) [5]. From a subjective perspective, workers can have the intention to quit or plan to quit when searching for occupational alternatives [6]. Research shows that nurses tend to experience higher rates of SA and turnover behaviors and intention compared with workers in other sectors [1, 2]. These “withdrawal behaviors” [7] affect nurses, healthcare organizations, and society. They can negatively impact employee motivation and job satisfaction [8] and result in direct and indirect costs for healthcare organizations [9]. They can also compromise the quality of patient care by disrupting service delivery [10, 11]. Thus, it is crucial to prevent work-related health problems that lead to nurses' SA and to make nurses' work environments conducive to retention.

SA and turnover behaviors and intention are influenced by factors pertaining to the individuals and their work environment [12]. It is worth mentioning that SA and turnover behaviors and intention are also impacted by societal factors such as legislation on Occupational Health and Safety (OHS) [12], but healthcare have little control over those factors. Individual factors include mental health, which is defined as a state characterized by the absence of symptoms and an ability to function and to flourish in one's work and community [13, 14]. This definition implies that mental health encompasses negative (e.g., symptoms of mental health problems) and positive (e.g., work engagement) dimensions [15, 16]. Mental health among nurses is a critical concern in the healthcare sector, as evidenced by the high prevalence of certain mental health problems. For instance, the global prevalence of burnout in nurses is estimated at 11.2% [17]. This poses a significant organizational challenge, given that SA due to mental health reasons tends to last longer than that related to physical health [18]. Moreover, the health-related determinants of nurse turnover behaviors and intention appear to be predominantly psychological, such as job dissatisfaction and burnout, rather than physical, according to a review of reviews [19]. Workers are also exposed to work-related psychosocial factors (WRPFs) that can have positive and negative effects on mental health, SA, and turnover behaviors and intention. Work-related psychosocial risk factors are aspects of the design and management of work (e.g., workload and work schedule), and its social and organizational contexts (e.g., workplace bullying and poor communication) that can cause psychological or physical harm [20]. Work-related psychosocial protective factors, such as social support and decision latitude, can reduce or prevent the negative impacts of risk factors. In nurses, prospective studies have shown that the risk of SA, turnover rates, and turnover intention was positively predicted by workplace bullying and negatively predicted by perceived social support from coworkers [21–25]. The Job Demands–Resources (JD–R) model [16] describes the direct and indirect correlations between WRPFs, mental health, SA, and turnover behaviors and intention [26]. This model posits that job demands (e.g., work-related psychosocial risk factors) can contribute to the development of certain mental and physical health problems, which can in turn increase the risk of SA and turnover behaviors and intention [26]. In contrast, job resources (e.g., work-related psychosocial protective factors) can attenuate the impact of risk factors on mental health problems [26]. The JD–R model also postulates that job resources can reduce SA and turnover behaviors and intention through an increase in work engagement [26].

Most preventive interventions of mental health problems, SA, and turnover behaviors and intention in nurses target nurses' individual factors, such as physical activity and mindfulness [27, 28]. With more countries requiring employers to address WRPFs in their OHS policies [29], it is imperative to support OHS stakeholders, including practitioners in nursing management and leadership, in selecting interventions that improve the exposure to WRPFs, employee mental health, and the functioning of healthcare organizations. In a risk management perspective, workplace interventions can reduce or eliminate the risk factors directly or increase the protective factors (i.e., primary prevention) [30, 31]. Workplace interventions can also help workers actively handle work-related psychosocial risk factors (i.e., secondary prevention) [30, 31]. However, there is a lack of consensus on which interventions both improve the exposure to WRPFs and reduce SA and turnover behaviors and intention in nurses, as well as on how to evaluate the effectiveness of these interventions. Previous reviews of interventions to promote nurses' mental health found only a minority of interventions that target the work environment and drew their conclusions on interventions that mainly targeted individual protective factors such as mindfulness [28]. Other reviews identified effective interventions to prevent SA and turnover [32, 33], but they only included controlled evaluation designs. Alternative study designs, such as pre–post, can be used to evaluate the effectiveness of interventions to reduce SA and turnover, but they have been overlooked in those reviews. This review focuses on interventions that target WRPFs to prevent SA and turnover behaviors and intention in nurses. This overarching purpose is divided into three specific objectives: (a) identify interventions that target WRPFs to reduce SA and turnover behaviors and intention in nurses; (b) describe the methods used to evaluate their effectiveness; and (c) report on their effectiveness at improving SA, turnover rates, and turnover intention in nurses.

## 2. Materials and Methods

A systematic search strategy was developed using the Preferred Reporting Items for Systematic Reviews and Meta-Analyses (PRISMA) Checklist [34]. The protocol was registered in PROSPERO (ID CRD42024567706). The PICO framework [35] was used to identify the following key concepts: (1) nurses; (2) workplace interventions; (3) WRPFs; (4) mental health; and (5) SA, turnover behaviors, or turnover intention.

### 2.1. Study Selection

Eight search engines and online databases were used (i.e., CINAHL, Embase, Catalogue ISST, Google Scholar, OSH Update, PsycINFO, PubMed, and Social SciSearch). A list of search terms was developed to capture each key concept. The list of search terms reflecting WRPFs was based on constructs featured in theoretical models that link WRPFs to occupational health (e.g., the JD–R model [16, 26]) and in validated questionnaires that measure WRPFs (e.g., the Copenhagen Psychosocial Questionnaire and the General Nordic Questionnaire [36, 37]). The lists of search terms were combined and applied in the “Title,” “Abstract,” and “Keywords” search fields (see the Supporting Information for the full syntaxes (available here)). The searches were conducted between February 21 and March 19, 2024, and restricted to documents published from 2001 onwards.

To be eligible, studies had to (1) involve nurses and/or nurse assistants (≥ 50% of the sample); (2) focus on an intervention acting on at least one WRPF; (3) report the results of an evaluation of the intervention's effectiveness at reducing the rates of SA, rates of turnover, or turnover intention in nurses; (4) employ a randomized controlled trial (RCT), quasi-RCT, cluster RCT, or pre–post design; (5) be peer-reviewed; and (6) be published in English or French. Eligible interventions had to target at least one WRPF (i.e., reducing a work-related psychosocial risk factor or promoting a work-related psychosocial protective factor). For the purpose of this review, workplace interventions are defined as specific actions or activities occasioning changes in the workplace, the work organization, or the job situation [38]. Studies were excluded if they focused on student nurses who are not part of the nursing workforce or aimed to help nurses withstand work-related psychosocial risk factors without correcting them (e.g., through bolstering mindfulness). Literature reviews, protocols, and documents from the gray literature were also excluded.

Based on the inclusion and exclusion criteria, a selection grid was developed and applied to all titles and abstracts. The first and the second authors applied the grid independently on a random sample of records, which constituted 50% of the whole corpus. Interrater reliability was assessed, and a Cohen κ statistic of 0.84 was obtained, which is considered strong [39]. Then, the eligibility of the selected studies was verified by applying the inclusion and exclusion criteria to the full articles.

### 2.2. Data Extraction, Synthesis, and Methodological Quality Assessment

A coding scheme was developed and applied to the articles included for review to meet each specific objective. To reach the first objective, the information related to the interventions' characteristics was extracted (i.e., name of the intervention, goal in relation to WRPFs, content, and format). Then, based on its goal and content, each intervention was categorized at a level of action following the IGLO (i.e., individual, group, leader, and organization) model [40]. This model suggests that employee mental health and behaviors are determined by factors located at the individual (e.g., skill utilization), group (e.g., colleagues support and work group climate), leader (e.g., managers' attitudes, behaviors, and support), and organization (e.g., human resources management practices and policies, job design, and occupational health services) levels.

To reach the second objective, the information related to the methods used to evaluate the effectiveness of the interventions was extracted (i.e., study design, control, follow-up, setting, sample size, and methods used to measure the outcomes). Based on this information, the methodological quality of the included studies was assessed using the Medical Education Research Study Quality Instrument (MERSQI) [41]. This tool contains 10 elements classified into the following six domains: (1) study design; (2) sampling; (3) type of data; (4) validity evidence for assessment instrument scores; (5) data analysis; and (6) outcome type. Each domain has a maximum score of three. For this review, the appraisal of the three latter domains was applied to the methods related to the evaluation of the effect of the intervention on the main outcomes (i.e., SA, and turnover behaviors, and intention). For the “outcome type” domain, a score of three was granted to studies that reported rates of SA or turnover, and a score of one was granted to those that used turnover intention only. The fourth domain was only applicable to studies that assessed psychological constructs, such as turnover intention, using self-report tools (e.g., questionnaires, scales, and indices). The studies that did not use such tools were given the mark “not applicable” for this domain. Thus, the maximum total MERSQI score for a study was either 18 (i.e., when all the domains were applicable) or 15 (i.e., when the fourth domain was not applicable). To facilitate comparison across studies, the total score was reported in percentages. The first and the second author applied the grid independently on all articles. Interrater reliability was assessed, and the percent agreement was 82.5%.

To reach the third objective, the results related to changes in SA rates, turnover rates, and turnover intention were extracted. This review considers WRPFs and mental health as mediators between the interventions and these three outcomes. Thus, results on changes in exposure to WRPFs (e.g., workplace bullying, civility, and leadership) and mental health (e.g., distress, burnout, work engagement, and job satisfaction) were also extracted when reported in the primary studies. Meta-analysis was not possible due to heterogeneity in intervention types as well as measures of SA and turnover behaviors and intention.

## 3. Results

**Figure 1:**
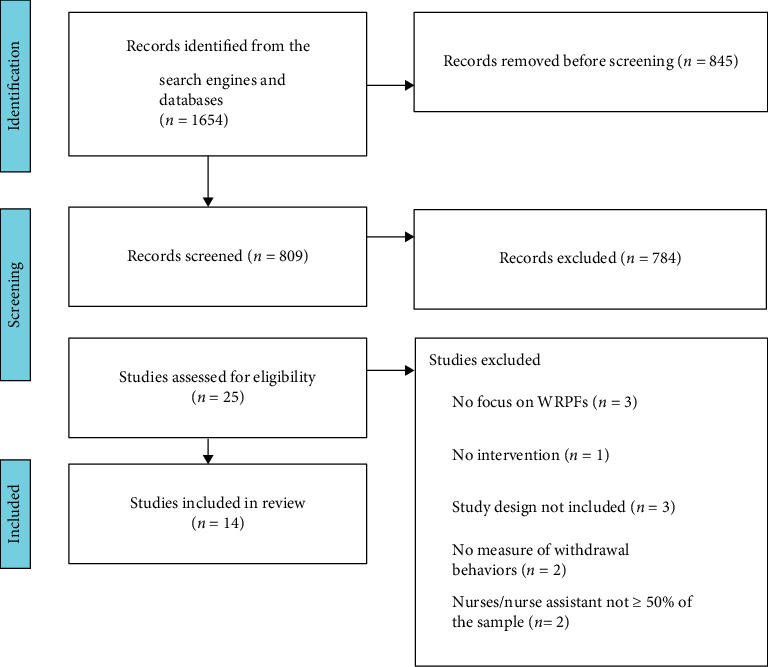
PRISMA flow diagram.

The searches yielded 1654 records. After combining the databases and removing duplicates, 809 records remained. The screening of the titles and abstracts led to the selection of 25 studies. The main reasons for exclusion were study design that did not include at least two measurement points and the absence of SA, turnover behaviors, or intention as an outcome. Fourteen studies based on 13 interventions were included after reading the full texts (see Figure 1).

### 3.1. Objective 1: Characteristics of the Interventions

This section describes the goals and contents of the interventions categorized in each of the IGLO levels. As indicated in Table 1, four interventions fostered nurses' skills in handling certain work-related psychosocial risk factors and were therefore located at the *individual level* [42, 44, 45, 56]. Nurses were exposed to education and training on how to handle situations of misconduct at work, such as workplace bullying and lateral violence (i.e., a set of negative behaviors produced between adults, such as workplace bullying, incivility, and social acts of disrespect [56]). Two interventions consisted in cognitive rehearsal for workplace bullying [57], an approach based on the theoretical assumptions of cognitive behavioral therapy (CBT). CBT is a counseling approach designed to solve individual problems by examining the relationships between thoughts, feelings, and behaviors [58].

Two interventions focused on group dynamics and were located primarily at the *group level.* The *Civility*, *Respect*, *Engagement in the Workplace* (CREW) was the focus of two studies [47, 48]. The CREW training fosters civility between colleagues through group discussions and activities. It is based on Fredrickson' broaden and build theory, which suggests that stress narrows employees' thinking and limits creative problem solving, while supportive work environments foster psychological safety, empowering employees to trust, grow, and perform at their best [59]. Following the *Practice Partnership Model of Care*, patient-centered and quality-focused initiatives (e.g., clinical handover at the bedside) were implemented to improve teamwork [46].

At the *leader level*, two interventions focused on improving the leadership of nurses and nurse managers. These included the *University of Pittsburgh Medical Center Leadership Development for Nursing Middle Managers* [49] and an ethical leadership program [50]. The interventions included individual and group activities based on education and training.

Five interventions that modified human resources management practices or job design were categorized at the *organizational level*. Two of them were exclusively related to schedules (i.e., self-scheduling and fixed schedules) [52, 55]. Four studies evaluated the effects of interventions that simultaneously targeted a range of elements of work organization in the healthcare sector. The *Cultural Change Toolkit* was implemented in an emergency department in one study [51]. Specifically, this intervention consists of a set of tools regarding literature-based measures that encourage positive practice changes. This intervention was designed to target mainly shared decision making, recognition, feedback, and communication. Based on the JD–R model [15, 16], another intervention consisted in a range of organizational measures (e.g., staffing and access to continuing professional development) to reduce workload and increase possibilities for development [53]. One intervention was centered around *Health circles*, a participatory approach to tackle adverse emergency department work conditions such as lack of personal breaks during work time, high-pressure environment, lack of staff information, and staff shortages [54].

### 3.2. Objective 2: Evaluation Methods

#### 3.2.1. Study Designs and Settings

Table 2 shows that most studies used a pre–post design (*n* = 8). Other types of designs included quasiexperimental (*n* = 3), cluster quasiexperimental (*n* = 1), and randomized control trial (*n* = 1). One study employed a mixed methods designs combining a pre–post assessments and qualitative interviews [54].

The studies were conducted in the United States (*n* = 5), South Korea (*n* = 3), Australia (*n* = 2), Canada (*n* = 2), and Europe (*n* = 2). The interventions were implemented in single organizations such as hospitals or medical centers (*n* = 5), multiple hospitals within hospital systems (*n* = 3), and units or departments within one (*n* = 3) or more hospitals (*n* = 2).

#### 3.2.2. Outcomes and Mediators Measured

SA was the outcome of four studies. Two measured the number of self-report SA episodes in the past month [47, 48], and the other two used administrative data to calculate the rate of SA (e.g., the ratio of lost hours to hours worked in the past 6 months) [46, 52]. The duration of SA ranged from 1 [47, 48] to 14 days of work [52]. Turnover rates were documented in six studies, mainly using administrative data on annual external turnover rates [42, 43, 49, 52, 53, 55]. Turnover intention was among the outcomes of six studies. It was measured using validated tools in four studies [44, 45, 47, 51] and a single item in two [50, 54]. Additionally, mediators included mental health symptoms (e.g., burnout, depressive symptoms, and psychological distress), work engagement (e.g., job satisfaction, organizational commitment, and work engagement), and WRPFs (e.g., social support, job control, supervisor leadership, and workplace bullying). These outcomes were almost exclusively measured using validated, self-report tools. The list of instruments to measure WRPFs and mental health is accessible upon request to the corresponding author.

#### 3.2.3. Methodological Quality Evaluation

As shown in Table 3, the average MERSQI score was 66.74% (SD = 12.99). The weakest domains were response rate (*M* = 1.50/3) and validity of self-report evaluation instruments to assess turnover intention (*M* = 1.57/3), and the strongest domains were the outcome type (*M* = 2.28/3) and data analysis (*M* = 2.57/3) (data not shown).

### 3.3. Objective 3: Intervention Effects on Outcomes and Mediators

At the *individual level*, turnover rates decreased in settings where interventions targeting lateral violence were implemented [42, 43]. Interventions that target workplace bullying were associated with short-term (i.e., 4 weeks after the beginning of the interventions) reductions in turnover intention. However, turnover intention scores did not differ statistically between baseline and 8 weeks after the beginning of the interventions [44, 45]. This type of intervention was also associated with improvements in the perceived quality of interpersonal relationships and a reduction in the severity of workplace bullying [44, 45] (see Table 2).

At the *group level*, CREW participants reported a greater decrease in the number of recent SA occasions compared with nonparticipants after 1 year [47], but this effect was not maintained after 2 years [48]. There was a nonsignificant reduction in SA rates following the implementation of the *Practice Partnership Model of Care* [46]. The CREW participants also benefited more than the control group regarding burnout severity, psychological distress, and job satisfaction. Group-level interventions were also associated with improvements in some WRPFs, such as increased civility and incivility among coworkers and supervisors [47, 48], as well as reduced frequency of nurse call bells, which is an objective indicator of workload [46].

At the *leader level*, one intervention was associated with a significant reduction in turnover rates, especially for newly hired nurses [49], and one did not significantly reduce turnover intention [50]. In one study, leadership education was associated with a reduction in some indicators of mental health, such as job satisfaction and commitment to the workplace, and occupational citizenship behaviors [50]. This type of intervention was associated with an increase in supervisors' perception of their own leadership [49, 50]. However, staff nurses perceived their supervisors' leadership less favorably after the intervention in one study [50].

At the *organizational level*, one intervention did not significantly change SA and turnover rates [52]. Two interventions were implemented in more than one organization at a time [53, 55]. The studies show that an intervention can be associated with different changes in turnover rates across sites (i.e., decrease, no significant change, or increase). Turnover intention was not impacted by the *Cultural Change Toolkit* [51]. Turnover intention also increased after a participatory intervention [54]. Organizational interventions generally had positive effects on mental health at work (e.g., depersonalization and emotional exhaustion) and WRPFs (e.g., job control), but job satisfaction decreased in one study [54].

## 4. Discussion

This systematic review identified 13 interventions aimed at improving the quality of nurses' psychosocial work environment to reduce SA and turnover behaviors and intention. Using the IGLO model [40], it was possible to show that interventions can target WRPFs by acting on individuals, groups of colleagues, leaders, and organizations.

At the individual level, four interventions aimed to reduce workplace bullying and lateral violence through education and training. The high prevalence of workplace bullying and lateral violence among nurses [62] may make these WRPFs a priority for researchers and OSH practitioners, which could explain why a relatively high proportion of interventions included in this review targeted these WRPFs. Regarding the contents of the interventions, the results are in line with those of a previous systematic review that showed that education and training are the preferred means of helping nurses cope with workplace bullying and lateral violence [63]. Only one group-level intervention was identified. Considering nurses' complex and demanding tasks, increasing teamwork and supportive working relationships through group activities could help reduce the negative impacts of these work-related demands on nurses' health, SA, and turnover behaviors and intention [64]. Two training-based interventions improved nurse managers' leadership. Education and training are the most widespread means of improving nurse leadership [65]. Interventions at the organizational level had various contents. Previous literature reviews showed that organizational interventions can take a variety of forms, including job redesign, schedule modification, and participatory interventions [27, 28, 66].

Although some interventions were associated with positive effects on the psychosocial work environment (i.e., reducing psychosocial risk factors and increasing the protective ones) and mental health (e.g., reducing mental health symptoms and increasing work engagement), few significantly reduced SA and turnover rates and intention. Most studies did not detect significant changes, and some even reported deteriorations. Differences in goals and contents of the interventions and in the methods used to evaluate their effectiveness could explain the variability in the results.

First, the goals and contents of most interventions included in this review were specific to a certain WRPF and level of intervention of the IGLO model, with some exceptions [51]. However, SA and turnover behaviors and intention are multidermined, and studies have shown that the accumulation of WRPFs increases their risk [21–25]. Targeting certain WRPFs may not suffice to reduce SA and turnover behaviors and intention in nurses. Relatedly, the interventions analyzed in this review might not have been successful at improving factors that mediate the relationship between WRPFs and SA and turnover behaviors and intention, such as mental health. The JD–R model states that WRPFs can influence SA and turnover behaviors and intention indirectly through health impairment and motivational processes [26]. Of the studies included in this review, the majority did not assess or report changes in mental health. When mental health was measured, the changes were not always significant. There is little data supporting the effectiveness of interventions that target the psychosocial work environment at improving mental health, particularly in reducing mental health symptoms. Previous meta-analyses showed that targeting WRPFs is associated with reductions in symptoms of burnout, depression, and psychological distress that are overall small in effect size or nonsignificant [67–70]. In contrast, there is more evidence on the effectiveness of interventions that target individuals' protective factors such as optimism and stress management abilities [67]. Individual's protective factors can reduce the negative impact of work-related psychosocial risk factors on employee mental health and increase the access to work-related psychosocial protective factors, and vice versa [26]. Comprehensive interventions that target both WRPFs and individual resources might be appropriate for the prevention of SA and turnover [70], but the evidence is still scarce.

Second, the use of follow-up measurements was heterogeneous across studies. Follow-up times ranged from 5 weeks [44] to 1.5 years [42] after the end of an intervention, and most studies used a pre–post design only without a follow-up. At the individual level, all four interventions reduced turnover rates and intention at post-test, but follow-up measures showed that the effects on turnover intention were not sustained over time. This aligns with a meta-analysis showing that stress management interventions focused on individual resources can generate measurable effects on stress reduction in the short term but that these effects may fade shortly after [27]. Considering the health impairment and motivational processes of the JD–R model, it is possible that targeting WRPFs with activities focusing on groups, leaders, and organizations generate impacts on SA and turnover behaviors and intention that take longer to occur and last longer than interventions that target individuals' skills at handling WRPFs. Moreover, in this review, reductions in SA and turnover rates were only detected 1 year after the implementation of the interventions. In some studies where the rates of SA and turnover were monitored more than 1-year postimplementation, gradual decreases were recorded [42, 49, 53]. This suggests that, to detect changes in SA and turnover rates, measurements should begin 1 year after the implementation of interventions on WRPFs and that annual follow-ups may be necessary.

Third, most studies did not use a control group. Control groups in evaluation studies increase the possibility for making a causal inference on interventions and outcomes [71]. When no significant changes are detected, an intervention might have had beneficial effects and prevented a deterioration in certain outcomes. Healthcare organizations frequently undergo contextual changes, such as service restructuring, governance and management changes, as well as layoffs, which influence nurses' occupational health, SA, and turnover behaviors and intention. These elements can counterbalance the beneficial effects of an intervention, and SA and turnover behaviors and intention may remain stable over time, making the intervention appear ineffective. When significant changes are reported, the attribution to the intervention is uncertain in the absence of comparisons between exposed and nonexposed individuals. Alternative hypotheses may explain the results. An example is regression to the mean, which could apply to some of the studies reviewed. Four of the included studies, none of which were controlled, were conducted in settings that were particularly affected by turnover before the interventions were implemented [42, 43, 49, 51]. It should be noted that, in the context of workplace intervention studies, certain organizational constraints may prevent the use of control groups [71], particularly in primary prevention. By focusing on the psychosocial work environment over individual resources, primary prevention simultaneously impacts all employees within the intervention setting. This type of intervention typically is directed at entire departments or organizations, rather than focusing on single individuals, and identifying a suitable comparison group for evaluation can be particularly difficult [72].

Fourth, the measurements of SA and turnover behaviors and intention differed across studies, with some using self-report tools, and others relying on objective indicators such as administrative data. In the methodological quality assessment, studies that used self-report tools were granted fewer points for the “Outcome type” domain than studies that used objective indicators (see Table 2). However, in the context of research on SA and turnover behaviors and intention in nurses, self-report data are not necessarily less valid than administrative data. A meta-analysis showed that self-report and administrative-based measures of SA generally converge [73]. Among nurses, turnover intention is a strong predictor of actual turnover over the course of a year [74, 75]. This aligns with the Theory of Planned Behavior [76, 77], which posits that individuals' attitudes lead to behavioral intentions (“turnover intentions” in this review), which in turn predict human behaviors such as leaving a job or an organization. In addition, when evaluating the effectiveness of organizational interventions at reducing SA and turnover rates, the use of administrative is flawed when the data are collected at the organization level. It is unlikely that all employees are equally exposed to an intervention that acts on WRPFs. Workers who are more exposed to these interventions may benefit more than their less-exposed colleagues. In this context, using the administrative data restricted to workers who enrolled in the study might be more appropriate [78]. Overall, combining sources of data on SA and turnover behaviors and intention is indicated [79].

### 4.1. Implications for Research

The main WRPFs under study were leadership, civility, lateral violence, workplace bullying, workload, low social support, decision authority, and lack of opportunities for professional development. These factors are relevant as they increase or decrease, depending on the factor, the risk of SA and turnover behaviors and intention in nurses [21, 22, 80, 81]. Other WRPFs that are particularly important do not appear in the literature reviewed. For example, most nurses report having been exposed to physical violence at least once [82]. In healthcare workers, this exposure is associated with the prevalence of mental health conditions, such as post-traumatic stress disorder, major depression, generalized anxiety disorder, and panic disorder, as well as SA and turnover [83–85]. More research is needed on the effectiveness of interventions that target workplace physical violence at reducing SA and turnover behaviors and intention in nurses.

Regarding the outcomes, it is worth mentioning that none of the studies used internal turnover as an outcome. Thus, there is no evidence to demonstrate that the interventions had an impact on this behavior. Moreover, none of the studies specified whether the episodes of SA were attributable to mental or physical health issues. This absence of detail currently prevents a clear understanding of whether the interventions that target WRPFs are more effective in addressing a specific type of health condition (i.e., mental or physical) that causes SA in nurses.

The results of this review show that some aspects of the interventions (i.e., the contexts and mechanisms that influence the outcomes) must be examined in future evaluation studies of SA and turnover prevention in nurses. First, this review showed that some contextual variables related to nurses and to interventions characteristics can contribute to more efficient interventions for certain groups. For instance, one intervention showed greater success in reducing turnover behaviors among newly recruited nurses [49]. Another study found that stronger implementation was linked to increased perceptions of civility following the intervention [47]. Additionally, two interventions showed varying impacts on turnover behaviors depending on the implementation setting [53, 55]. These results show that certain contextual factors pertaining to the participants (e.g., length of experience as a nurse) and to the delivery of the intervention (e.g., implementation strength and setting) may influence the effectiveness of the interventions at reducing SA and turnover behaviors and intention in nurses. It is worth mentioning that the influence of variables such as sex and gender was not examined in the included papers. However, a literature review reports that within employment groups where the distribution of sex or gender is uneven, employees belonging to the minority group experience higher rates of SA [86]. This may be attributed to disparities in the perception of demands and resources [86]. Such findings pave the way for future research focusing on differences in the effectiveness of interventions based on sex or gender, given the predominance of women in this profession [87].

Second, considering the mechanisms and the contribution of the realistic approach, there is little evidence to validate the presumed mechanisms that underpin the association between intervention targets (i.e., WRPFs) and outcomes (i.e., SA and turnover behaviors and intention). Some studies did not track changes in WRPFs, and only a minority assessed changes in mental health [44, 47, 48, 50, 53, 54]. This is in line with a recent literature review which showed that, among prospective cohort studies on the longitudinal effect of exposure to WRPFs on the incidence of SA, few investigated the mediators involved [88]. It is possible that an intervention reduces SA and turnover behaviors and intention without improving nurses' mental health, for example, by increasing presenteeism via a motivational pathway [89]. Measuring intermediate outcomes would ensure that the mechanism of an intervention is the one that operates. Relatedly, only four out of thirteen interventions were based on a theoretical model of human behavior. Thus, future interventions should be more firmly grounded in theory, and evaluation research might benefit from adopting a realistic stance. Realistic evaluation identifies the contexts and mechanisms that enable the success of an intervention. The underlying research questions go as follows: “What works, for whom, in what respects, to what extent, in what contexts, and how?” [90]. Adopting this approach would make it possible to identify the subgroups of nurses that benefit more from the interventions and to determine how correcting WRPFs is associated with mental health, SA and turnover behaviors and intention [91].

### 4.2. Implications for Practice

The heterogeneity in the goals and contents of the interventions and in the methods used to evaluate their effectiveness makes it unclear what needs to be put in place to address SA and turnover behaviors and intention by targeting WRPFs in nurses. However, interventions aimed primarily at improving the relational aspect of the psychosocial work environment, in particular individual interventions designed to train nurses on how to handle situations of workplace bullying and lateral violence, appear to reduce turnover rates and intention, albeit in the short term. Enhancing interventions with refresher activities could help maintain the effects over time.

To promote mental health at work in nurses, it is essential to shape healthy psychosocial work environments. Involving nurses in decision-making is crucial in achieving this [92], which makes participatory approaches relevant (e.g., Participatory Action Research) [93]. However, some participatory interventions in the healthcare sector were associated with negative effects on turnover intention, burnout, engagement, job satisfaction, workload, and perceived social support [54, 78, 94]. These effects may be explained by barriers to the implementation of participatory interventions such as staff shortages [95], the limited time and financial resources to take part in the activities and to stay informed [54, 94, 96], the increased effort required to execute the action plans [95, 97], the lack of organizational support [78, 96], the difficulty in reaching common understandings [94], and the insecurity and role conflicts caused by work reorganizations [78]. Practitioners in nursing management and leadership may address these barriers by developing action plans composed of strategies that are simple to implement or distributed and sequenced in a realistic period [78, 98], bringing together management and employees with a shared goal of identifying problems and solutions [95, 96], and exchanging views and good practices with key persons of other units at group meetings [94].

### 4.3. Limitations

This review has some limitations that open avenues for further research. First, it was limited to interventions that target WRPFs, whereas SA and turnover behaviors and intention in nurses stem from a host of individual and organizational factors. A further review could broaden the criteria to all types of interventions that ultimately aim at reducing SA and turnover behaviors and intention in nurses. Second, this review did not consider documents from the gray literature such as reports by public agencies. It is possible that some organizations in the healthcare sector have implemented interventions targeting WRPFs and evaluated their effectiveness in reducing SA and turnover behaviors and intention. For example, antibullying policies are common practice in the healthcare sector [99], and reports may have documented changes in SA and turnover rates associated with the implementation of such policies. A future review of the gray literature could identify effective interventions to reduce SA and turnover that were not identified in this research. Overall, the inclusion criteria led to a small number of studies that focused on a wide range of interventions, and the results should be interpreted with caution.

## 5. Conclusions

The current state of the literature does not make it possible to indicate with certainty which WRPFs to focus on and how to target them to prevent SA and turnover behaviors and intention in nurses. To help nursing managers select interventions that help reduce SA and turnover behaviors and intention in their organizations, and to respond to nurses' demands for improved quality of the work environment, both the content of interventions and the methods used to assess their effectiveness must be improved. Research should focus on WRPFs and intervention contents that nurses prioritize, notably by increasing the participation of nurses in the development, implementation, and evaluation of the interventions. Evaluation protocols could be improved by including control groups and follow-up measures that allow detecting the stability of the effects of interventions on nurses' psychosocial environment and SA and turnover behaviors and intention and by investigating the contexts and mechanisms that make intervention effective or not. By improving the quality and depth of research in this area, researchers and practitioners in nursing management and leadership will be better equipped to implement strategies that not only enhance nurses' mental health but also contribute to the overall stability and performance of healthcare organizations.

## Figures and Tables

**Table 1:** Characteristics of the 13 interventions evaluated in the 14 included studies, by level of intervention.

Intervention [reference]	Goal in relation to WRPFs	Content	Format
Individual level			
Workshops on lateral violence [42]	To enhance assertive communication skills and raise awareness about the impact of lateral violence	Workshops on communication training focused on healthy conflict resolution and eliminating a culture of silence for nurses	Duration (total): N/R Duration (session): 60–90 min Frequency: Once Participants: Nurse staff and managers
Cognitive rehearsal program [43]	To improve nurses' abilities to effectively combat lateral violence	Education and training on lateral violence	Duration (total): 2 h Duration (session): 2 h Frequency: N/R Participants: Nurse staff and managers
Cognitive rehearsal program [44]	To provide nurses with personal strategies to cope with workplace bullying	Education and training on workplace bullying and nonviolent communication	Duration (total): 5 weeks Duration (session): 2 h Frequency: Twice a week Participants: Nurses and charge nurses
Cognitive rehearsal program [45]	To provide nurses with personal strategies to cope with workplace bullying	Education and training on workplace bullying and nonviolent communication delivered through a mobile application	Duration (total): 8 weeks Duration (session): N/A Frequency: N/A Participants: General staff nurses
Group level			
Practice Partnership Model of Care [46]	To improve teamwork	A model of patient care that includes working in partnership, clinical handover at the bedside, comfort rounds, and environmental modifications	Duration (total): March 2008 to June 2008 Duration (session): N/A Frequency: N/A Participants: Nurse staff
Civility, Respect, and Engagement in the Workplace Leiter et al. [47] and Leiter et al. [48]	To improve civility	Group training focused on improving colleague relationships	Duration (total): 6 months Duration (session): N/R Frequency: Weekly Participants: Healthcare workers
Leader level			
The University of Pittsburgh Medical Center Leadership Development for Nursing Middle Managers [49]	To develop leadership in nurse managers	Education and training on leadership	Duration (total): 2 months Duration (session): 8 h Frequency: 5 sessions Participants: Nurse managers
Ethical leadership program [50]	To help university hospital nursing unit managers to understand ethical leadership and foster ethical climates in their units and organizations	Training on ethical leadership	Duration (total): 6 months Duration (session): 2 h Frequency: Monthly Participants: Nurse managers
Organization level			
Cultural Change Toolkit [51]	To improve the perception of the clinical practice environment (i.e., meaningful recognition, shared decision making, and increased leadership support and involvement)	Information and tools regarding measures that encourage positive practice changes (e.g., a department-specific gratitude board, a thank-you card program for both staff and leadership use, a practice-based suggestion box, daily leadership rounding, and daily feedback)	Duration (total): 2 months Duration (session): N/A Frequency: N/A Participants: Nurse staff and leaders
Fixed scheduling [52]	To implement fixed scheduling	Use of fixed schedules with day and evening shifts. Nurses are allowed to change and trade shifts	Duration (total): N/A Duration (session): N/A Frequency: N/A Participants: Nurse staff
Workload intervention [53]	To reduce workload	A range of workload reduction measures including a nursing workload tool; assessment of nursing workloads; increase in nurse staff; increased access to clinical supervision and support for graduates; increased access to continuing professional development	Duration (total): 5 years Duration (session): N/A Frequency: N/A Participants: Nurse staff, managers, and directors
Health circles [54]	To change emergency departments work factors (e.g., lack of personal breaks during work time, high-pressure environment, lack of staff information, and staff shortages)	A participatory intervention to tackle adverse emergency department work conditions	Duration (total): 7 months Duration (session): 90 min Frequency: 10 sessions Participants: Nurses and physicians
Self-scheduling [55]	To implement self-scheduling	A self-scheduling intervention that allows nurses to select their schedule	Duration (total): N/A Duration (session): N/A Frequency: N/A Participants: Nurses

Abbreviations: N/A, not applicable; N/R, not reported; US, United States; WRPFs, work-related psychosocial factors.

**Table 2:** Study design, outcomes, and results, by level of intervention.

Reference	Study design, control group type, and measurement time	Setting and sample size at pre- and post-test	SA and turnover behaviors and intention measurement	Results on SA and turnover behaviors and intention	Results on mental health	Results on WRPFs
Individual level						
Ceravolo et al. [42]	Pre–post CG: N/A T1: 4 years	A five-hospital integrated healthcare delivery system (Northeastern US) NPre: 703 NPost: 485	Type: Turnover rate Source: Administrative data Measure: Unclear Time: Unclear	Turnover rate decreased from 8.9% to 6.0% 3 years after the workshops were implemented	N/A	Decreases in some indicators of lateral violence (significance was not tested)
Embree et al. [43]	Pre–post CG: N/A T1: 0.5–1 year T2 2 years	A critical access hospital (Indiana, US) NPre: 48 NPost: 35	Type: Turnover rate Source: National database Measure: Proportion of nurses voluntarily separating from the organization Time: 12 months	Turnover rate decreased from 7.84% before implementation to 1.42% after 1 year	N/A	N/A
Kang et al. [44]	Randomized controlled trial CG: Waitlist T1: 5 weeks T2: 9 weeks	A university hospital (B city, South Korea) NPre: 44 NPost: 40 NFU: 19	Type: Turnover intention Source: Self-report Measure: Intent to quit the organization (Korean version of the *Intent to quit* scale) [60]	Turnover intention at postintervention decreased in the IG only. Nonsignificant follow-up effect in the IG	Nonsignificant group × time effect on psychological distress	Greater improvement in perceived quality of interpersonal relationships in the IG than in the CG Nonsignificant group × time effect on workplace bullying
Kang and Jeong [45]	Cluster quasirandomised design CG: No intervention T1: 4 weeks T2: 8 weeks	A university hospital (Busan, South Korea) NPre: 73 NPost: 72 NFU: 72	Type: Turnover intention Source: Self-report Measure: Intent to quit the organization (Korean version of the *Intent to quit* scale) [60]	Turnover intention decreased more in the IG than in the CG from baseline to 4 weeks of possessing the app—not after 8 weeks	N/A	Perceived person‐related bullying and work-related bullying decreased more in the IG than in the CG Nonsignificant group × time effect on intimidation-related bullying
Group level						
Cann and Gardner [46]	Pre–post CG: N/A T1: 9 months T2: N/A	An acute surgical ward (Northern half of Queensland, Australia) NPre: N/R NPost N/R	Type: Sickness absence rate Source: Administrative data Measure: Number of hours lost divided by number of hours worked Time: 6 months	Nonsignificant decrease in SA (i.e., from 11%; in October 2007–March 2008 to 7% in June 2008–November 2008)	N/A	Decrease of nurse call bells frequency Nonsignificant increases in patient complaints and compliments
Leiter et al. [47]	Quasiexperimental CG: No intervention T1: 1 year T2: N/A	Eight units from five hospitals (Nova Scotia and Ontario, Canada) NPre: 1173 NPost: 907	Type: Sickness absence Source: Self-report Measure: Number of occasions where the participant missed work due to illness or disability Time: 1 month Type: Turnover intention Source: Self-report Measure: Three items based on Kelloway, Gottlieb, and Barham (1999)	Six months after the implementation period, improvement was greater in the IG than the CG only for SA, not turnover intention Nonsignificant group × time interaction effect for turnover intention after the intervention Interaction effect suggests a significant decrease in past-month sickness absence rate in IG only	Greater improvements in cynicism and job satisfaction in the IG compared to the CG Nonsignificant group × time effect on professional efficacy and emotional exhaustion	Greater improvements in perceived colleague civility, supervisor incivility, respect, and management trust in the IG compared with the CG Nonsignificant group x time effect on colleague incivility and instigated incivility Stronger implementation was associated with more perceived civility after the intervention
Leiter et al. [48]	Quasiexperimental CG: No intervention T1: 1 year T2: 2 years	Eight units from five hospitals (Nova Scotia and Ontario, Canada) NPre: 957 NPost: 680 NFU: 643	Type: Sickness absences Source: Self-report Measure: Number of occasions where the participant missed work due to illness or disability Time: 1 month	The effect of the intervention on past-month self-report SA was not maintained 1 year after the intervention	Greater improvements in psychological distress, civility, and supervisor incivility in the IG compared with the CG	No change in coworker incivility and instigated incivility
Leader level						
Fennimore and Wolf [49]	Pre–post CG: N/A T1: 6 months T2: N/A	The University of Pittsburgh Medical Center Pittsburgh (Pittsburgh, US) NPre: 22 NPost: 21	Type: Turnover rate Source: Administrative data Measure: Unclear Time: 12 months	Turnover rate decreased from 10.07% in 2006 to 9.2% in 2009 (all nurses), and from 17% in 2006 to 11% in 2009 (new nurse and new graduate nurses)	N/A	Increases in management and leadership abilities (significance was not tested)
Jeon et al. [50]	Pre–post CG: N/A T1: 6 months T2: N/A	A tertiary university hospital (Seoul, South Korea) NPre: 200 NPost: 158	Type: Turnover intention Source: Self-report Measure: Single yes/no item on the intention to leave the hospital	Nonsignificant decrease in turnover intention in staff nurses at postintervention	*Nurse staff* Decreases in occupational citizenship behaviors, commitment to workplace, and job satisfaction	*Unit managers* Increased perception of self-report people orientation and concern for sustainability. Ethical leadership only improved in unit managers with < 5 years of managing experience No effect on task responsibility fairness, relationship fairness, power sharing, ethical guidance, and integrity *Nurse staff* Decrease in perceived ethical leadership of unit managers Improvement on perceived influence at work No effect on justice and respect, horizontal trust, and vertical trust
Organization level						
Adams et al. [51]	Pre–post CG: N/A T1: 2 months T2: N/A	An emergency department at a community hospital (Southeast Texas, US) NPre: 38 NPost: 32	Type: Turnover intention Source: Self-report Measure: The degree to which the nursing staff member perceived they would terminate their position eventually at some unspecified time in the future (*Anticipated turnover scale*) [61]	No effect on turnover intention at postintervention	N/A	N/A
Kullberg et al. [52]	Quasiexperimental CG: self-scheduling T1: 8 months T2: N/A	Four adult oncological inpatient wards (Sweden) NPre: 92 NPost: 101	Type: Sickness absence rate Source: Administrative data Measure: Percentage of short-term SA (< 15 days) out of scheduled working time Time: 1 month Type: Turnover rate Source: Administrative data Measure: Number of nursing staff being employed or quitting their job during the divided by the average number of employed staff Time: 12 months	No apparent effect of fixed scheduling on turnover rates and short-term SA (< 15 days) after 8 months	N/A	Increased general working conditions and team spirit and decreased overtime and changes of shift at short notice
Rickard et al. [53]	Pre–post CG: N/A T1 2 years T2: N/A	Two hospitals (Northern Territories, Australia) NPre: 178 NPost: 206	Type: Turnover rate Source: Archival data Measure: Turnover rate of nurses and midwives in each hospital Time: 1 month	Significant decrease in turnover rate in one of the two hospitals (i.e., from 46% in May 2004 to 28% in June 2008 and 29% in June 2010)	Decreased psychological distress and emotional exhaustion and increased job satisfaction	Decreased job demands and increased supervisor support, coworker support, job control, flexible/adaptable culture, psychosocial safety climate, and communication within the organization Nonsignificant effects on consultation and preparation for the job and opportunity for professional development
Schneider et al. [54]	Pre–post and qualitative interviews CG: N/A T1: 7 months T2: N/A	An interdisciplinary emergency department of a tertiary referral hospital (Southern Germany) NPre: 76 NPost: 73	Type: Turnover intention Source: Self-report Measure: Single item	Increased turnover intention over 1 year	Decreased depersonalization and job satisfaction No effects on emotional exhaustion and depressive symptoms	Increase in job control and decreased social support and number of overtime hours No effect on participation opportunities, work overload, personnel resources, information problems, uncertainty, and supervisor feedback Qualitative interviews indicate that three participants perceived that patient number and staffing worsened after the intervention
Wright et al. [55]	Pre–post CG: N/A T1: 3 years T2: N/A	A four-hospital system (Southeastern US) NPre: 1317 NPost: 1492	Type: Turnover rate Source: Administrative data Measure: External turnover rate of registered nurses in each hospital Time: 12 months	Three years after implementation, nurse turnover rates increased in two hospitals and decreased in two hospitals	N/A	Perceived autonomy increased in all four hospitals, and perceived professional development increased in three hospitals

*Note:* FU: follow-up time after the end of the intervention; NFU: sample size at follow-up; NPre: sample size at pretest; NPost: sample size at post-test.

Abbreviations: CG, control group; IG, intervention group; N/A, not applicable; N/R, not reported; SA, sickness absence; WRPFs, work-related psychosocial factors.

**Table 3:** Methodological quality evaluation of the included studies, by level of intervention.

Study	Study design	Sampling		Type of data	Validity of evaluation instrument			Data analysis			Total
		Number of organizations	Response rate		Internal structure	Content validity	Relationships to other variables	Sophistication	Appropriate	Outcome type	
Individual level											
Ceravolo et al. [42]	1.5	0.5	0.5	3	N/A	N/A	N/A	1	1	3	70.0%
Embree et al. [43]	1.5	0.5	0.5	3	N/A	N/A	N/A	1	1	3	70.0%
Kang et al. [44]	3	1	0.5	1	1	1	1	2	1	1	69.4%
Kang and Jeong [45]	2	0.5	1.5	1	1	1	1	2	1	1	66.7%
Group level											
Cann and Gardner [46]	1.5	0.5	0.5	3	N/A	N/A	N/A	2	1	3	76.7%
Leiter et al. [47]	2	1.5	0.5	1	1	1	1	2	1	3	77.8%
Leiter et al. [48]	2	1.5	0.5	1	0	0	0	2	1	3	61.1%
Leader level											
Fennimore and Wolf [49]	1.5	0.5	0.5	3	N/A	N/A	N/A	1	1	3	70.0%
Jeon et al. [50]	1.5	0.5	1.5	1	0	0	0	1	1	1	41.7%
Organization level											
Adams et al. [51]	1.5	0.5	0.5	1	1	0	1	2	1	1	52.8%
Kullberg et al. [52]	2	0.5	1.5	3	N/A	N/A	N/A	1	1	3	80.0%
Rickard et al. [53]	1.5	1	0.5	3	N/A	N/A	N/A	2	1	3	80.0%
Schneider et al. [54]	1.5	0.5	0.5	1	0	0	0	2	1	1	41.7%
Wright et al. [55]	1.5	1.5	0.5	3	N/A	N/A	N/A	1	1	3	76.7%

Abbreviation: N/A, not applicable.

## Data Availability

Data and materials have not been made publicly available elsewhere, as all the data and materials are included in the tables, figures, and supporting information.

## Supporting Information

The Supporting Information include the complete lists of search terms, by search engine and online database.
