# Supplementary material for: Improving the Psychosocial Work Environment to Prevent Sickness Absence and Turnover in Nurses: A Systematic Review
Source: J Nurs Manag. 2025 Oct 22;2025:7860752. doi: 10.1155/jonm/7860752 (PMC12571538; doi:10.1155/jonm/7860752)
Supplement: Supporting Information — Additional supporting information can be found online in the Supporting Information section. [file 7860752.f1.docx]

**SEARCH QUERIES**

**Manuscript** Improving the psychosocial work environment to prevent sickness absence and turnover in nurses: A systematic review

**Author names and affiliations** Corthésy-Blondin, Laurent^1^; Negrini, Alessia^1^; Vila Masse, Samantha^1^; Genest, Christine^2,3^

^1^ Institut de recherche Robert-Sauvé en santé et en sécurité du travail, 505 boul. De Maisonneuve Ouest Montréal (Québec) H3A 3C2
^2^ Faculty of Nursing, Université de Montréal, 2375, chemin de la Côte-Ste-Catherine Montréal (Québec) H3T 1A8
^3^ Centre de recherche de l’Institut universitaire de santé mentale de Montréal, 7401 Rue Hochelaga, Montréal (Québec) H1N 3M5

**Corresponding authoe** [laurent.corthesy-blondin@irsst.qc.ca](mailto:laurent.corthesy-blondin@irsst.qc.ca)

**Catalogue ISST**

"Personnel infirmier" / Sujet

AND

"Absentéisme maladie" OR "Mobilité de la main-d'oeuvre" / Sujet

**EMBASE +CINAHL**

"Nurse*" OR "Nursing assistant*" OR "Nursing personnel" OR "Nursing staff" / TIAB

AND

"Behavior change" OR "Behaviour change" OR "behavior modification" OR "behaviour modification" OR Implementation OR Implemented OR Improvement OR Improving OR Initiative OR Intervention* **OR** "mental health program*" OR "occupational health measure*" OR "Occupational measure*" OR "organisational change" OR "Organisational intervention*" OR "organisational policy" OR "organisational practice*" OR "Organisational measure*" OR "organizational change" OR "Organizational intervention*" OR "organizational policy" OR "organizational practice*" OR "Organizational Measure*" OR "policy making" OR Prevention OR "workplace intervention*" OR "workplace program*" / TIAB

AND

("Social support" AND (work OR colleagues OR supervisor* OR manager OR leader* OR organizational OR organizational)) **OR** (Feedback AND (leader* OR performance OR supervisor* OR work)) OR Effort NEAR/1 reward OR Traumatic NEAR/0 event OR

Workplace NEAR/2 violence OR Autonomy OR Bullying OR "Cognitive demands" OR Control OR "Decision latitude" OR "effort-reward*" OR "Emotional demands" OR "emotional workload" OR "empowering leadership" OR "fair leadership" OR harass* OR "human resource primacy" OR "Influence at work" OR "innovative climate" OR "job control" OR "Job demands" OR "Job insecurity" OR "job Precarity" OR "Job resources" OR "Job satisfaction" OR "leadership quality" OR "learning demands" OR "learning opportunities" OR "Long working hours" OR "Mental workload" OR "Organisational injustice" OR "Organizational injustice" OR "possibilities for development" OR Predictability OR "Psychological demand*" OR "Psychosocial factor*" OR "Psychosocial risk factor*" OR "Quality of life at work" OR "role clarity" OR "role conflict" OR "security at work" OR "social climate" OR strain OR stress OR Stressful OR Stressor* OR "task variety" OR "Temporary employment" OR "Work intensification" OR "work pace" OR "work-family balance" OR "work-family conflict" OR "work-health balance" OR "Work-life imbalance" OR Workload OR "work-load" OR "work-to-family spillover" / TIAB

AND

substance NEAR/1 abuse OR substance NEAR/1 use OR "acute stress" OR addiction* OR "adjustment disorder*" OR "affective disorder*" OR "Alcohol use" OR anxiety OR "burn out" OR burnout OR "burn-out" OR "common mental disorder*" OR cynicism OR depersonalization OR depression OR disability OR "Drug use" OR "emotional exhaustion" OR "emotional trauma" OR "health status" OR Insomnia OR "mental disorder*" OR "mental health" OR "mental health problem*" OR "Occupational disease" OR "occupational health" OR "posttraumatic stress" OR "Psychological distress" OR "psychological health" OR "Psychological health" OR PTSD OR "sleep disorder*" OR "work related illness*" / TIAB

AND

"absence*" OR "absenteeism" OR "sick leave" OR "sickleave" OR "sick-leave" OR "sickness absence" OR (Intent NEAR/1 leave OR Intention NEAR/1 leave OR Intent NEAR/1 quit OR Intention NEAR/1 quit OR "Leaving practice" OR "Leaving profession" OR "Personnel retention" OR Retaining OR Turnover*) NOT (Cigarette OR Mitochondrial OR Smoker* OR Smoking OR Tobacco) / TI

**OSH Update**

Nurse OR "Nursing assistant" OR "Nursing personnel" OR "Nursing staff" / Abstract

AND

"absence*" OR "absenteeism" OR "sick leave" OR "sickleave" OR "sick-leave" OR "sickness absence" OR Intent OR leave OR Intention OR Leaving OR Quit OR Quitting OR Retention OR Retaining OR Turnover*

NOT (Cigarette OR Mitochondrial OR Smoker* OR Smoking OR Tobacco) / Abstract

**PSYCINFO**

"Nurse*" OR "Nursing assistant*" OR "Nursing personnel" OR "Nursing staff" / TIAB

AND

"Behavior change" OR "Behaviour change" OR "behavior modification" OR "behaviour modification" OR Implementation OR Implemented OR Improvement OR Improving OR Initiative OR Intervention* **OR** "mental health program*" OR "occupational health measure*" OR "Occupational measure*" OR "organisational change" OR "Organisational intervention*" OR "organisational policy" OR "organisational practice*" OR "Organisational measure*" OR "organizational change" OR "Organizational intervention*" OR "organizational policy" OR "organizational practice*" OR "Organizational Measure*" OR "policy making" OR Prevention OR "workplace intervention*" OR "workplace program*" / TIAB

AND

("Social support" AND (work OR colleagues OR supervisor* OR manager OR leader* OR organizational OR organizational)) **OR** (Feedback AND (leader* OR performance OR supervisor* OR work)) OR Effort NEAR/1 reward OR Traumatic NEAR/0 event OR

Workplace NEAR/2 violence OR Autonomy OR Bullying OR "Cognitive demands" OR Control OR "Decision latitude" OR "effort-reward*" OR "Emotional demands" OR "emotional workload" OR "empowering leadership" OR "fair leadership" OR harass* OR "human resource primacy" OR "Influence at work" OR "innovative climate" OR "job control" OR "Job demands" OR "Job insecurity" OR "job Precarity" OR "Job resources" OR "Job satisfaction" OR "leadership quality" OR "learning demands" OR "learning opportunities" OR "Long working hours" OR "Mental workload" OR "Organisational injustice" OR "Organizational injustice" OR "possibilities for development" OR Predictability OR "Psychological demand*" OR "Psychosocial factor*" OR "Psychosocial risk factor*" OR "Quality of life at work" OR "role clarity" OR "role conflict" OR "security at work" OR "social climate" OR strain OR stress OR Stressful OR Stressor* OR "task variety" OR "Temporary employment" OR "Work intensification" OR "work pace" OR "work-family balance" OR "work-family conflict" OR "work-health balance" OR "Work-life imbalance" OR Workload OR "work-load" OR "work-to-family spillover" / TIAB

AND

substance NEAR/1 abuse OR substance NEAR/1 use OR "acute stress" OR addiction* OR "adjustment disorder*" OR "affective disorder*" OR "Alcohol use" OR anxiety OR "burn out" OR burnout OR "burn-out" OR "common mental disorder*" OR cynicism OR depersonalization OR depression OR disability OR "Drug use" OR "emotional exhaustion" OR "emotional trauma" OR "health status" OR Insomnia OR "mental disorder*" OR "mental health" OR "mental health problem*" OR "Occupational disease" OR "occupational health" OR "posttraumatic stress" OR "Psychological distress" OR "psychological health" OR "Psychological health" OR PTSD OR "sleep disorder*" OR "work related illness*" / TIAB

AND

"absence*" OR "absenteeism" OR "sick leave" OR "sickleave" OR "sick-leave" OR "sickness absence" OR Intent NEAR/1 leave OR Intention NEAR/1 leave OR Intent NEAR/1 quit OR Intention NEAR/1 quit OR "Leaving practice" OR "Leaving profession" OR "Personnel retention" OR Retaining OR Turnover*

NOT Cigarette OR Mitochondrial OR Smoker* OR Smoking OR Tobacco

/ TI

**PUBMED**

22774352[PMID] OR 23066695[PMID]

"Nurse*"[Title/Abstract] OR "Nursing assistant*"[Title/Abstract] OR "Nursing personnel"[Title/Abstract] OR "Nursing staff"[Title/Abstract] (1)

AND

"Behavior change"[TIAB] OR "Behaviour change"[TIAB] OR "behavior modification"[TIAB] OR "behaviour modification"[TIAB] OR Implementation[TIAB] OR Implemented[TIAB] OR Improvement[TIAB] OR Improving[TIAB] OR Initiative[TIAB] OR Intervention*[TIAB] **OR** "mental health program*"[TIAB] OR "occupational health measure*"[TIAB] OR "Occupational measure*"[TIAB] OR "organisational change"[TIAB] OR "Organisational intervention*"[TIAB] OR "organisational policy"[TIAB] OR "organisational practice*"[TIAB] OR "Organisational measure*"[TIAB] OR "organizational change"[TIAB] OR "Organizational intervention*"[TIAB] OR "organizational policy"[TIAB] OR "organizational practice*"[TIAB] OR "Organizational Measure*"[TIAB] OR "policy making"[TIAB] OR Prevention[TIAB] OR "workplace intervention*"[TIAB] OR "workplace program*"[TIAB] (2)

AND

"Social support"[TIAB] AND (work[TIAB] OR colleagues[TIAB] OR supervisor*[TIAB] OR manager[TIAB] OR leader*[TIAB] OR organizational[TIAB] OR organizational[TIAB]) (3)

OR

Feedback[TIAB] AND (leader*[TIAB] OR performance[TIAB] OR supervisor*[TIAB] OR work[TIAB]) (4)

OR

"Effort reward"[Title/Abstract:~1] (5)

OR

"Traumatic event"[Title/Abstract:~0] (6)

OR

"Workplace violence"[Title/Abstract:~2] (7)

OR

Autonomy[TIAB] OR Bullying[TIAB] OR "Cognitive demands"[TIAB] OR Control[TIAB] OR "Decision latitude"[TIAB] OR "effort-reward*"[TIAB] OR "Emotional demands"[TIAB] OR "emotional workload"[TIAB] OR "empowering leadership"[TIAB] OR "fair leadership"[TIAB] OR harass*[TIAB] OR "human resource primacy"[TIAB] OR "Influence at work"[TIAB] OR "innovative climate"[TIAB] OR "job control"[TIAB] OR "Job demands"[TIAB] OR "Job insecurity"[TIAB] OR "job Precarity"[TIAB] OR "Job resources"[TIAB] OR "Job satisfaction"[TIAB] OR "leadership quality"[TIAB] OR "learning demands"[TIAB] OR "learning opportunities"[TIAB] OR "Long working hours"[TIAB] OR "Mental workload"[TIAB] OR "Organisational injustice"[TIAB] OR "Organizational injustice"[TIAB] OR "possibilities for development"[TIAB] OR Predictability[TIAB] OR "Psychological demand*"[TIAB] OR "Psychosocial factor*"[TIAB] OR "Psychosocial risk factor*"[TIAB] OR "Quality of life at work"[TIAB] OR "role clarity"[TIAB] OR "role conflict"[TIAB] OR "security at work"[TIAB] OR "social climate"[TIAB] OR strain[TIAB] OR stress[TIAB] OR Stressful[TIAB] OR Stressor*[TIAB] OR "task variety"[TIAB] OR "Temporary employment"[TIAB] OR "Work intensification"[TIAB] OR "work pace"[TIAB] OR "work-family balance"[TIAB] OR "work-family conflict"[TIAB] OR "work-health balance"[TIAB] OR "Work-life imbalance"[TIAB] OR Workload[TIAB] OR "work-load"[TIAB] OR "work-to-family spillover"[TIAB] (8)

AND

"substance abuse"[Title/Abstract:~1] (10)

"substance use"[Title/Abstract:~1] (11)

"acute stress"[TIAB] OR addiction*[TIAB] OR "adjustment disorder*"[TIAB] OR "affective disorder*"[TIAB] OR "Alcohol use" OR anxiety[TIAB] OR "burn out"[TIAB] OR burnout[TIAB] OR "burn-out"[TIAB] OR "common mental disorder*"[TIAB] OR cynicism[TIAB] OR depersonalization[TIAB] OR depression[TIAB] OR disability[TIAB] OR "Drug use"[TIAB] OR "emotional exhaustion"[TIAB] OR "emotional trauma"[TIAB] OR "health status"[TIAB] OR Insomnia[TIAB] OR "mental disorder*"[TIAB] OR "mental health"[TIAB] OR "mental health problem*"[TIAB] OR "Occupational disease"[TIAB] OR "occupational health"[TIAB] OR "posttraumatic stress"[TIAB] OR "Psychological distress"[TIAB] OR "psychological health"[TIAB] OR "Psychological health"[TIAB] OR PTSD[TIAB] OR "sleep disorder*"[TIAB] OR "work related illness*"[TIAB] (12)

AND

"absence*"[TIAB] OR "absenteeism"[TIAB] OR "sick leave"[TIAB] OR "sickleave"[TIAB] OR "sick-leave"[TIAB] OR "sickness absence"[TIAB] (14)

OR

"Intent leave"[Title/Abstract:~1] (15)

OR

"Intention leave"[Title/Abstract:~1] (16)

OR

"Intent quit"[Title/Abstract:~1] (17)

OR

"Intention quit"[Title/Abstract:~1] (18)

OR

"Leaving practice"[TIAB] OR "Leaving profession"[TIAB] OR "Personnel retention"[TIAB] OR Retaining[TIAB] OR Turnover*[TIAB] (19)

NOT

Cigarette[TIAB] OR Mitochondrial[TIAB] OR Smoker*[TIAB] OR Smoking[TIAB] OR Tobacco[TIAB] (20)

(1) AND (2) AND (3 OR 4 OR 5 OR 6 OR 7 OR 8) AND (10 OR 11 OR 12) AND ((14 O OR 15 OR 16 OR 17 OR 18 OR19) NOT (20))

**SOCIAL SCISEARCH**

"Nurse*" OR "Nursing assistant*" OR "Nursing personnel" OR "Nursing staff" / TIAB

AND

"Behavior change" OR "Behaviour change" OR "behavior modification" OR "behaviour modification" OR Implementation OR Implemented OR Improvement OR Improving OR Initiative OR Intervention* **OR** "mental health program*" OR "occupational health measure*" OR "Occupational measure*" OR "organisational change" OR "Organisational intervention*" OR "organisational policy" OR "organisational practice*" OR "Organisational measure*" OR "organizational change" OR "Organizational intervention*" OR "organizational policy" OR "organizational practice*" OR "Organizational Measure*" OR "policy making" OR Prevention OR "workplace intervention*" OR "workplace program*" / TIAB

AND

("Social support" AND (work OR colleagues OR supervisor* OR manager OR leader* OR organizational OR organizational)) **OR** (Feedback AND (leader* OR performance OR supervisor* OR work)) OR Effort NEAR/1 reward OR Traumatic NEAR/0 event OR

Workplace NEAR/2 violence OR Autonomy OR Bullying OR "Cognitive demands" OR Control OR "Decision latitude" OR "effort-reward*" OR "Emotional demands" OR "emotional workload" OR "empowering leadership" OR "fair leadership" OR harass* OR "human resource primacy" OR "Influence at work" OR "innovative climate" OR "job control" OR "Job demands" OR "Job insecurity" OR "job Precarity" OR "Job resources" OR "Job satisfaction" OR "leadership quality" OR "learning demands" OR "learning opportunities" OR "Long working hours" OR "Mental workload" OR "Organisational injustice" OR "Organizational injustice" OR "possibilities for development" OR Predictability OR "Psychological demand*" OR "Psychosocial factor*" OR "Psychosocial risk factor*" OR "Quality of life at work" OR "role clarity" OR "role conflict" OR "security at work" OR "social climate" OR strain OR stress OR Stressful OR Stressor* OR "task variety" OR "Temporary employment" OR "Work intensification" OR "work pace" OR "work-family balance" OR "work-family conflict" OR "work-health balance" OR "Work-life imbalance" OR Workload OR "work-load" OR "work-to-family spillover" / TIAB

AND

substance NEAR/1 abuse OR substance NEAR/1 use OR "acute stress" OR addiction* OR "adjustment disorder*" OR "affective disorder*" OR "Alcohol use" OR anxiety OR "burn out" OR burnout OR "burn-out" OR "common mental disorder*" OR cynicism OR depersonalization OR depression OR disability OR "Drug use" OR "emotional exhaustion" OR "emotional trauma" OR "health status" OR Insomnia OR "mental disorder*" OR "mental health" OR "mental health problem*" OR "Occupational disease" OR "occupational health" OR "posttraumatic stress" OR "Psychological distress" OR "psychological health" OR "Psychological health" OR PTSD OR "sleep disorder*" OR "work related illness*" / TIAB

AND

"absence*" OR "absenteeism" OR "sick leave" OR "sickleave" OR "sick-leave" OR "sickness absence" OR (Intent NEAR/1 leave OR Intention NEAR/1 leave OR Intent NEAR/1 quit OR Intention NEAR/1 quit OR "Leaving practice" OR "Leaving profession" OR "Personnel retention" OR Retaining OR Turnover*) NOT (Cigarette OR Mitochondrial OR Smoker* OR Smoking OR Tobacco) / TI
